# Exploring the interrelationships between athlete burnout, depression, and anxiety: a network analysis approach

**DOI:** 10.3389/fpsyg.2026.1845637

**Published:** 2026-07-07

**Authors:** Yuantai Fu, Yong-Gwan Song, Renchun Liang, Jiaqi Wang, Junyan Zhao, Yuwen Shangguan, Taojing Zhuang

**Affiliations:** 1 Department of Marine Sports, Pukyong National University, Busan, Republic of Korea; 2 College of Physical Education and Sports, Hainan Normal University, Haikou, Hainan, China; 3 College of Elementary Education, Hainan Normal University, Haikou, Hainan, China; 4 Department of Exercise Physiology, Kunsan National University, Gunsan-si, Jeollabuk-do, Republic of Korea; 5 Department of Orthopaedics, Changzhou Maternal and Child Health Care Hospital, Changzhou Medical Center, Nanjing Medical University, Changzhou, China

**Keywords:** anxiety, athlete burnout, depression, mental health, network analysis

## Abstract

**Background:**

Athlete burnout frequently co-occurs with depression and anxiety, yet prior studies relying on total scores obscure how individual symptoms interact across these constructs.

**Objective:**

To identify central and bridge symptoms linking athlete burnout, depression, and anxiety using network analysis.

**Methods:**

A total of 1,226 Chinese collegiate athletes (637 male, 589 female; M_age_ = 18.11, SD = 1.39) completed the ABQ-15, PHQ-9, and GAD-7. A regularized partial-correlation network was estimated with the EBIC-LASSO; expected influence (EI) and bridge expected influence (bEI) indexed centrality.

**Results:**

GAD-2 (uncontrollable worry; EI = 1.07), PHQ-2 (depressed mood; EI = 1.22), and ABQ-E8 (physical exhaustion; EI = 1.11) were the most central nodes. PHQ-3 (sleep disturbance; bEI = 0.47) emerged as the strongest bridge across the three clusters. The strongest edges connected ABQ-E8–PHQ-4 (fatigue; r = 0.37) and ABQ-E2 (training fatigue)–PHQ-1 (anhedonia; r = 0.29).

**Conclusion:**

GAD-2, PHQ-2, and ABQ-E8 exhibited high centrality and represent key intervention targets for reducing the overall symptomatology of burnout–depression–anxiety comorbidity in athletes, and should therefore be prioritized; PHQ-3, as the strongest bridge symptom connecting the three clusters, represents a target whose treatment may interrupt the transdiagnostic symptom-transmission pathways that maintain the comorbidity. These clearly defined intervention targets also facilitate the development of a brief, multi-symptom screening tool tailored to athlete populations.

## 1 Introduction

Athlete burnout is a complex psychological syndrome triggered by chronic stress and is widely recognized as a critical threat to athletes’ mental health (Dišlere et al., 2025; Eklund and DeFreese, 2020). Mean burnout levels among athletes have risen over the past two decades (Madigan et al., 2022). Burnout undermines athletes’ well-being and interpersonal relationships, and contributes to performance decline and elevated injury risk (Woods et al., 2025). Moreover, burnout rarely occurs in isolation; it frequently co-occurs with depression and anxiety and may precipitate or exacerbate both (Gustafsson et al., 2017; Koutsimani et al., 2019). Yet despite this well-documented association, the mechanisms through which the three conditions are interlinked remain poorly understood, constraining the precision of early identification and intervention.

Depression and anxiety within competitive sports environments have similarly garnered increasing attention (Souter et al., 2018). Research has demonstrated that the prevalence of common mental health problems among athletes is comparable to or even higher than that observed in the general population (Gouttebarge et al., 2019). A large-scale, multi-year study involving NCAA Division I collegiate athletes found that the prevalence of clinically significant depressive symptoms (CES-D ≥ 16) was 23.7%, with 6.3% of athletes reporting moderate to severe depressive symptoms (Wolanin et al., 2016). Anxiety symptoms are equally prevalent among athlete populations, with research indicating that approximately one-third of athletes reported anxiety symptoms, while 19.6% experienced psychological distress (Rice et al., 2019). Depression and anxiety in athletes not only adversely affect athletic performance but may also result in injuries and delayed recovery. Given that athletes are chronically exposed to unique stressors—including injury risk, training load, competitive selection pressures, and multiple role demands—the identification of early indicators of depression and anxiety is of considerable importance for the timely provision of psychological support and intervention (Runacres and Marshall, 2024).

Notably, burnout does not exist in isolation; symptoms of depression and anxiety are closely intertwined with burnout, and these three constructs often form mutually influencing symptom clusters that collectively exert a substantial impact on athletes’ mental health (Koutsimani et al., 2019). The majority of existing research has examined the relationships among these three constructs based on questionnaire total scores or factor scores, generally supporting the trend that “higher levels of burnout are associated with more severe symptoms of depression and anxiety” (Gao and Wang, 2024). Furthermore, a meta-analysis has demonstrated stable associations between athlete burnout and multiple adverse mental health outcomes (Glandorf et al., 2025). However, such correlational evidence based on macro-level indicators remains insufficient to address a question that is more critical for mechanistic elucidation and intervention practice: Which specific symptoms serve as the connectors linking burnout, depression, and anxiety, and drive the maintenance or amplification of the comorbidity system?

The analytical methods commonly employed in comorbidity research are ill-suited to the specific question of which symptoms link the three syndromes of burnout, depression, and anxiety. First, regression analyses and structural equation models (SEM) built on total or factor scores typically model constructs as reflective latent factors (Borsboom et al., 2003). Within this framework, each syndrome is conceived as the common latent cause of its observable symptoms, and associations among constructs are estimated solely at the latent-variable level; the pathways linking individual symptoms are consequently subsumed within the latent factors, making them difficult to identify directly (Borsboom, 2008; Cramer et al., 2010). Equating a total score with syndrome severity, moreover, carries the implicit assumption that all symptoms are equally indicative of the underlying construct. Yet research on the heterogeneity of syndromes such as depression has called this assumption into question (Fried and Nesse, 2015). Second, person-centered approaches such as latent class analysis (LCA) and latent profile analysis (Leiter and Maslach, 2016; Mäkikangas and Kinnunen, 2016) partition a sample into subtypes according to individuals’ response patterns. Valuable as such methods are for typological inquiry, they delineate which individuals cluster together rather than which symptoms relate to one another, and thus they too leave unanswered the central question of how symptoms transmit influence across syndromes (Borsboom, 2017; Cramer et al., 2010).

Against this backdrop, Borsboom (2017) proposed the network theory, which posits that psychological variables directly influence one another rather than being caused by an unobserved latent factor. In other words, the mutual interactions among symptoms themselves constitute the mental disorder, rather than merely serving as external manifestations of an underlying disease entity. This theoretical perspective enables researchers to transcend traditional total score-based assessment approaches and gain deeper insight into the specific mechanisms through which these psychological phenomena interact. Symptom network analysis offers two significant advantages. First, this method enables the identification of central symptoms within the symptom network—symptoms that may play critical roles in maintaining or triggering the overall network structure (Borsboom and Cramer, 2013). Second, network analysis facilitates the detection of potential “bridge symptoms,” which are key nodes that serve connective and transmissive functions between different symptom clusters, representing potential risk pathways through which different psychological problems mutually influence and propagate (Borsboom et al., 2021). By identifying these central symptoms and bridge symptoms, researchers and clinical practitioners can prioritize them as intervention targets, thereby designing more targeted and precision-oriented psychological intervention programs.

To date, the application of network analysis in athlete populations remains at an early stage, with only a handful of studies having adopted the approach in athlete samples. Geiger et al. (2023) estimated a network of psychological distress, generalized anxiety, depression, and somatic symptom disorder in 275 German elite athletes, revealing associations among symptoms and identifying related sport-specific factors such as injury and training load; however, their study did not incorporate burnout and modeled symptoms at the scale rather than the item level, leaving fine-grained inter-symptom pathways obscured. Drawing on a regularized partial correlation network, Poulus et al. (2024) examined burnout, resilience, and coping among 453 esports players and found problem-focused and avoidant coping items to be the most influential nodes; yet this study omitted depression and anxiety symptoms, and its sample comprised esports players rather than traditional competitive athletes. In general and clinical populations, by contrast, network analysis has been widely applied to map the comorbidity of burnout, depression, and anxiety (Ernst et al., 2021; Wang et al., 2025; Verkuilen et al., 2021), identifying several central symptoms such as anhedonia and exhaustion. Taken together, although network analysis is attracting growing attention in athlete mental health research, no study has yet examined the symptom-level structure of the burnout–depression–anxiety triad in athletes. Given the well-documented co-occurrence of these three conditions, this constitutes a critical gap.

The present study aims to apply network analysis to estimate a symptom network of burnout, depression, and anxiety in athletes, in order to identify both the central symptoms that maintain this comorbid system and the bridge symptoms that connect distinct symptom clusters and may propagate comorbidity risk across them. In doing so, it seeks to provide empirical evidence for the early identification of at-risk athletes and for the development of more precise, mechanism-based interventions.

## 2 Methods

### 2.1 Participants and procedures

This study used a cross-sectional survey design, with data collected between June and August 2025. After obtaining approval from the institutional ethics review board, we used convenience sampling to recruit collegiate athletes from 16 universities in central and northeastern China. The inclusion criteria were as follows: (1) being currently enrolled as a full-time student at a Chinese university; (2) being a registered athlete who competed in collegiate sport on behalf of one’s university and engaged in systematic training at least five times per week; and (3) being actively involved in training or competition during the data collection period.

The questionnaire was administered online through an electronic survey platform.^1^ After receiving the survey link distributed by team administrators, participants were first presented with a detailed information page that outlined the study’s objectives, procedures, and potential risks and benefits. All participants were required to provide electronic informed consent before beginning the questionnaire, and all data were collected and stored anonymously to protect participants’ privacy and confidentiality.

A total of 1,307 questionnaires were received. After excluding 81 invalid responses (completion time less than 1 min), 1,226 valid responses were included in the final analysis (male = 637, female = 589; Mage = 18.11, SD = 1.39). All items in the online questionnaire were set as mandatory, which prevented participants from submitting incomplete responses. Consequently, no missing data were present in the final datase.

### 2.2 Measures

#### 2.2.1 Athlete Burnout Questionnaire (ABQ-15)

Athlete burnout was assessed using the 15-item Athlete Burnout Questionnaire (Raedeke and Smith, 2001), which evaluates three dimensions: emotional/physical exhaustion, reduced sense of accomplishment, and sport devaluation. In the present study, the ABQ-15 demonstrated high internal consistency (*α* = 0.78).

#### 2.2.2 Patient Health Questionnaire (PHQ-9)

Depressive symptoms were assessed using the PHQ-9, a widely utilized 9-item self-report measure (Kroenke and Spitzer, 2002) that requires participants to evaluate the frequency of depressive symptoms experienced over the preceding 2 weeks. In the present study, the PHQ-9 demonstrated high internal consistency (*α* = 0.83).

#### 2.2.3 Generalized Anxiety Disorder Scale (GAD-7)

Anxiety symptoms were assessed using the GAD-7, a 7-item self-report measure designed to assess the severity of generalized anxiety symptoms (Spitzer et al., 2006). In the present study, the GAD-7 demonstrated high internal consistency (*α* = 0.88).

### 2.3 Statistical analysis

#### 2.3.1 Network estimation

All analyses were conducted using R (version 4.1.1). We employed the goldbricker function from the R package networktools (version 1.3.0) to examine redundancy, which identifies item pairs with correlations significantly below the 25% threshold to exclude statistically redundant items (Jones et al., 2021).

We estimated the depression-anxiety-burnout symptom network structure using the graphical least absolute shrinkage and selection operator (LASSO) in combination with the Extended Bayesian Information Criterion (EBIC) for model selection (Chen and Chen, 2008; Epskamp et al., 2018). This procedure was implemented using the bootnet package (version 1.6.0) in R (Epskamp et al., 2018). The network was estimated from a correlation matrix rather than a covariance matrix; because correlations are scale-invariant, all variables were thereby placed on a common (standardized) scale, preventing items with larger variances from dominating the network. Given the ordinal nature of the Likert-scaled items, polychoric correlations were computed using the cor_auto function in qgraph (Epskamp et al., 2018), which served as input to the graphical LASSO. The LASSO method computes a regularized partial correlation network in which “edges” represent the unique associations between two symptoms after controlling for all other symptoms in the network. The EBIC tuning parameter was set to 0.5 to control for spurious edges, thereby balancing model sensitivity and specificity (Krämer et al., 2009). In the final visualized network, each symptom is represented as a “node.” Connections between nodes are “edges,” representing their partial correlation relationships. Edge thickness corresponds to the strength of the association, with thicker edges indicating stronger associations. Edge color indicates the direction of the association: blue edges represent positive associations, whereas red edges represent negative associations.

In network analysis, strength, closeness, and betweenness are commonly used centrality indices. However, recent research has indicated that for networks estimated using LASSO regularization in psychological research, path-based metrics such as closeness and betweenness may be unstable and fail to yield reliable results (Bringmann et al., 2019). Furthermore, traditional indices such as strength, which calculate the sum of absolute edge weights, may not accurately reflect a node’s true influence in networks containing both positive (activating) and negative (inhibiting) edges; expected influence is a more appropriate index in such cases (Robinaugh et al., 2016). Therefore, expected influence was employed as the centrality index in the present study. Additionally, to explore symptoms that connect the distinct symptom communities of burnout, depression, and anxiety, we conducted bridge centrality analysis. This analysis was implemented using the bridge function from the networktools package (version 1.4.0) in R (Jones, 2017). Because our aim was to identify bridge symptoms among three predefined disorder domains, network communities in the bridge centrality analysis were defined *a priori* according to the three measures, with each node assigned to one of three communities representing burnout (ABQ-15), depression (PHQ-9), or anxiety (GAD-7). This approach has precedent in prior cross-disorder studies of bridge symptoms (Jin et al., 2022; He et al., 2023). Specifically, we calculated the bridge expected influence for each node. This metric quantifies the extent to which a symptom within one community is directly associated with symptoms in other communities, thereby identifying the most critical pathways between these symptom clusters.

#### 2.3.2 Network stability and accuracy

To assess the robustness of the network structure, we utilized the bootnet package (version 1.4.3) in R (Epskamp et al., 2018). We examined the correlation stability coefficient (CS-coefficient) through a case-dropping bootstrap procedure and evaluated the accuracy of edge weights by estimating 95% confidence intervals. A CS-coefficient above 0.25 is considered acceptable, whereas values above 0.50 indicate excellent stability. Finally, the statistical significance of differences in network properties was evaluated using bootstrapped difference tests (Epskamp et al., 2018). This testing method determines whether statistically significant differences exist between network metrics such as edge weights and node strength, providing a reliable statistical foundation for quantitative comparisons of network structures.

## 3 Results

### 3.1 Network estimation structure and local network properties

Table 1 presents the descriptive statistics for individual items related to athlete burnout, depression, and anxiety symptoms. To ensure data quality and the reliability of subsequent analyses, we performed a data screening procedure prior to conducting the network analysis. Specifically, we examined the mean levels of each item, the amount of information (as indicated by item-level standard deviations), and potential item redundancy. The results showed that all items fell within acceptable informational thresholds-defined as being within 2.5 standard deviations (ABQ-15: MSD = 0.74 ± 0.18, PHQ-9: MSD = 0.65 ± 0.28, GAD-7: MSD = 0.67 ± 0.03) In addition, the goldbricker analysis identified no problematic item pairs: for every pair, the proportion of significantly different correlations with other nodes exceeded the 0.25 threshold, indicating no statistically redundant items. Accordingly, no items were merged or removed, and all 31 items were retained in the final network.

**Table 1:** Symptom means, standard deviations, and predictability.

Scale	Items	*Mean*	*SD*	Predictability
ABQ-15	I feel “wiped out” from sport.	1.53	0.92	0.39
	I feel so tired from my training that I have trouble finding energy to do other things.	1.60	0.89	0.23
	I feel overly tired from my sport participation.	1.53	0.75	0.17
	I feel physically worn out from sport	1.49	0.71	0.17
	I am exhausted by the mental and physical demands of sport	1.58	0.74	0.20
	I’m accomplishing many worthwhile things in sport.	1.51	0.73	0.16
	I am not achieving much in sport.	1.57	0.74	0.15
	I am not performing up to my ability in sport.	1.54	0.70	0.24
	It seems that no matter what I do, I do not perform as well as I should.	1.52	0.79	0.14
	I feel successful at sport.	1.52	0.72	0.19
	The effort I spend in sport would be better spent doing other things.	1.53	0.70	0.16
	I do not care as much about my sport performance as I used to.	1.52	0.70	0.16
	I’m not into sport like I used to be.	1.49	0.71	0.16
	I feel less concerned about being successful in sport than I used to.	1.54	0.69	0.14
	I have negative feelings toward sport.	1.51	0.69	0.15
PHQ-9	Little interest or pleasure in doing things.	0.57	0.77	0.42
	Feeling down, depressed, or hopeless.	0.47	0.63	0.35
	Trouble falling or staying asleep, or sleeping too much.	0.56	0.78	0.41
	Feeling tired or having little energy.	0.53	0.75	0.25
	Poor appetite or overeating.	0.45	0.64	0.34
	Feeling bad about yourself? Or that you are a failure or have let yourself or your family down.	0.51	0.62	0.34
	Trouble concentrating on things, such as reading the newspaper or watching television.	0.50	0.61	0.33
	Moving or speaking so slowly that other people could have noticed? Or the opposite—being so fidgety or restless that you have been moving around a lot more than usual.	0.42	0.61	0.13
	Thoughts that you would be better off dead or of hurting yourself in some way.	0.26	0.44	0.41
GAD-7	Feeling nervous, anxious, or on edge.	0.59	0.68	0.47
	Not being able to stop or control worrying.	0.57	0.65	0.43
	Worrying too much about different things.	0.58	0.66	0.40
	Trouble relaxing.	0.63	0.68	0.45
	Being so restless that it is hard to sit still.	0.60	0.68	0.39
	Becoming easily annoyed or irritable.	0.59	0.67	0.42
	Feeling afraid, as if something awful might happen.	0.61	0.67	0.44

**Figure 1:**
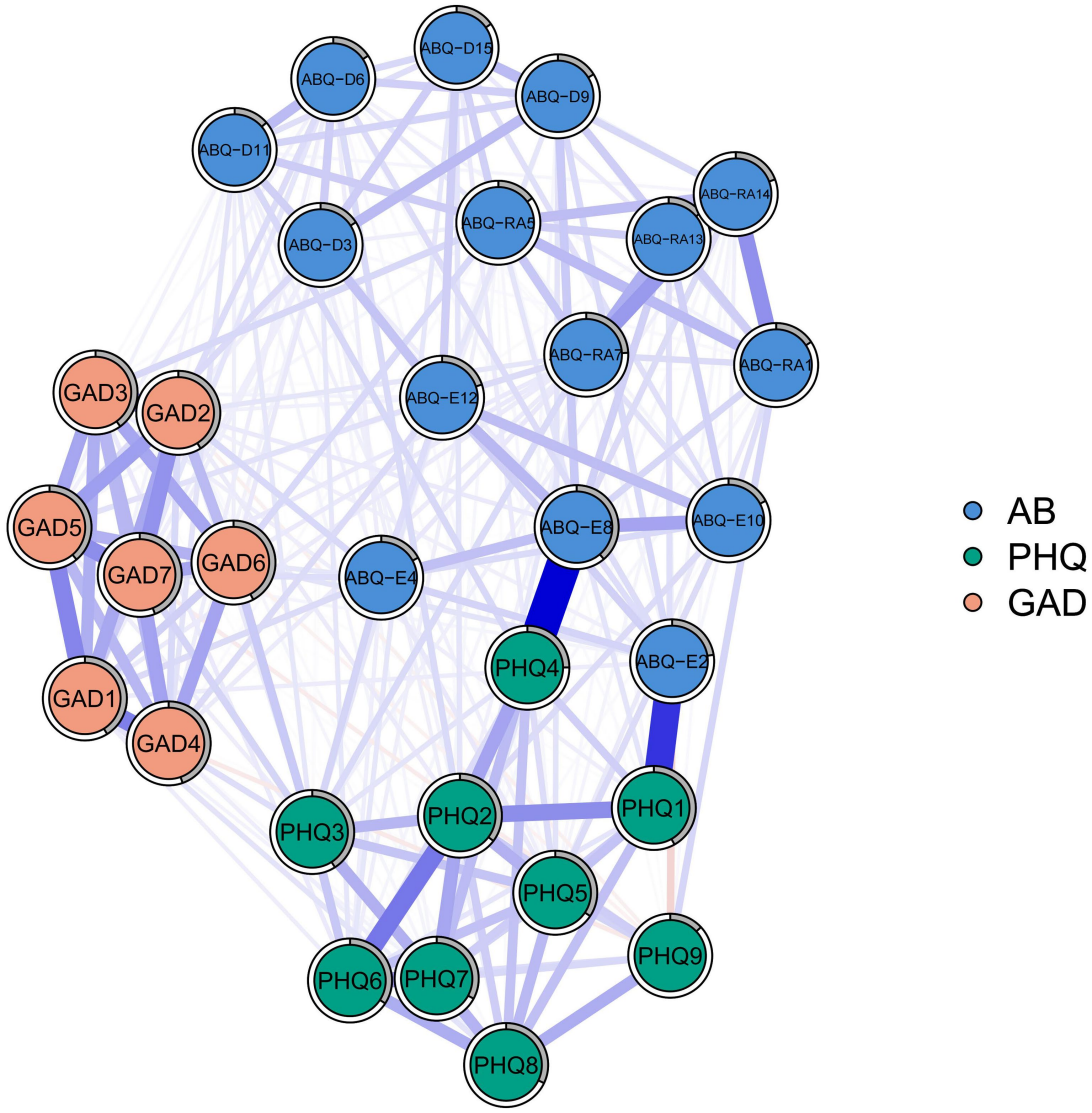
The regularized network among athlete burnout, depression, and anxiety symptoms.

**Figure 2:**
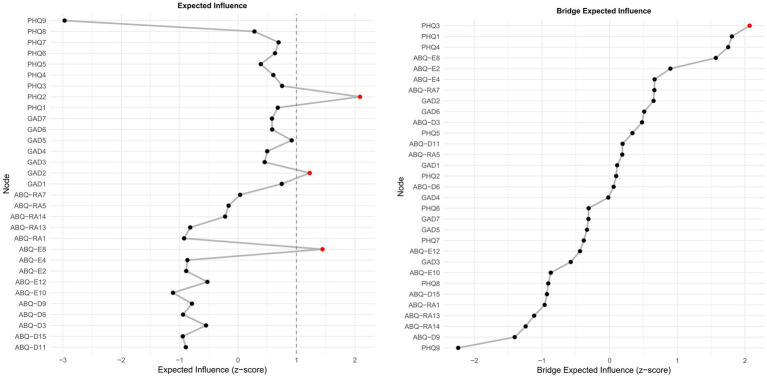
Expected influence **(A)** and bridge expected influence **(B)** centrality indices.

Figure 1 illustrates the symptom network estimated using the PHQ-9, GAD-7, and ABQ-15 scales. The resulting network was relatively dense, with 270 out of 465 possible edges being present. Examination of the weighted adjacency matrix (see Table S1) identified the strongest edge weights within the network. Specifically, the connection between ABQ-E8 (Physical exhaustion) and PHQ-4 (Fatigue) showed the highest edge strength, followed by the link between ABQ-E2 (training fatigue) and PHQ-1 (anhedonia). Furthermore, local node-level analysis based on the expected influence index revealed that PHQ-2 (depressed mood; EI = 1.22), ABQ-E8 (physical exhaustion; EI = 1.11), and GAD-2 (uncontrollable worry; EI = 1.07) had the highest expected influence scores in the network (see Figure 2A). These findings indicate that these three nodes occupy core positions in the symptom network, exerting the strongest direct and indirect influence on other symptoms.

### 3.2 Bridge nodes

Figure 2B displays the distribution pattern of bridge expected influence centrality. The analysis showed that PHQ-3 (Sleep disturbance; bEI = 0.47) ranked highest in bridge expected influence. To verify the robustness of this result, we conducted a case-dropping bootstrap analysis. The correlation stability coefficient for bridge expected influence was 0.595, which exceeds the commonly accepted threshold of 0.25, indicating satisfactory statistical stability of the bridge centrality metric (see Figure 2B).

### 3.3 Network stability and accuracy

**Figure 3:**
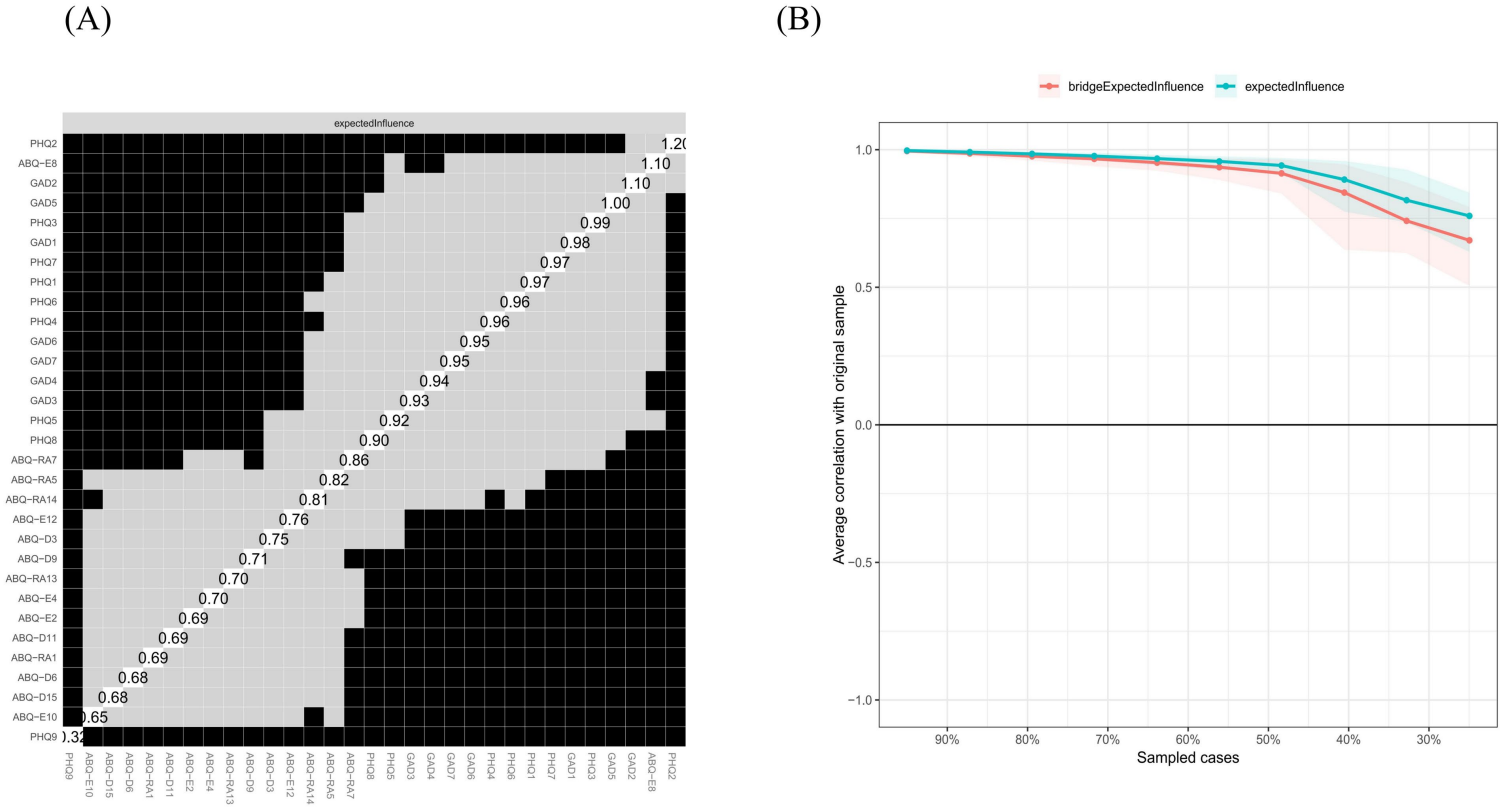
Bootstrapped difference test **(A)** and case-dropping stability **(B)** for centrality indices.

Edge weight stability analysis demonstrated a high degree of consistency between observed edge weight distributions and those generated via nonparametric bootstrap resampling. Strong edges, in particular, showed good reproducibility, supporting the robustness of the estimated network structure (see Figure 3A). Bootstrapped difference tests of edge weights further confirmed the statistical reliability of the network connections, with most pairwise comparisons reaching statistical significance (see Supplementary Figures S1, S2). The stability of the expected influence metric was evaluated using a case-dropping bootstrap procedure, which showed that expected influence remained stable across different levels of case removal. The correlation stability coefficient (CS-C) for expected influence was 0.67, which exceeds the 0.25 threshold for acceptable stability (see Figure 3B).

## 4 Discussion

Using network analysis, the present study estimated a comorbidity network of burnout, depression, and anxiety among Chinese collegiate athletes, yielding three principal findings. First, the nodes with the highest expected influence (EI) were PHQ-2 (depressed mood; EI = 1.22), ABQ-E8 (physical exhaustion; EI = 1.11), and GAD-2 (uncontrollable worry; EI = 1.07), indicating that these symptoms occupy central positions in the network. Second, PHQ-3 (sleep disturbance; bridge expected influence = 0.47) emerged as the key bridge node linking the three symptom clusters and may serve as an important conduit for cross-disorder risk transmission. Third, the strongest edges were observed between physical exhaustion and fatigue (ABQ-E8 and PHQ-4) and between training-related fatigue and anhedonia (ABQ-E2 and PHQ-1), reflecting symptom overlap in energy depletion and loss of interest. This contribution not only enriches the theoretical understanding within the field of athlete mental health but also responds to calls from transdiagnostic psychopathology research to explore the shared maintaining mechanisms across different psychological problems (Dalgleish et al., 2020; Fusar-Poli et al., 2019).

Regarding node centrality, GAD-2 (uncontrollable worry), PHQ-2 (depressed mood), and ABQ-E8 (Physical exhaustion) exhibited the highest expected influence (EI) indices, indicating that these symptoms possess the strongest overall connections with other symptoms within the burnout-depression-anxiety network. From a clinical practice perspective, these central symptoms warrant substantial attention, as prioritizing them in interventions may trigger more efficient cascading ameliorative effects throughout the network. Overall, this finding aligns with the transdiagnostic model’s proposition that different forms of psychological distress may share common maintaining factors, suggesting that symptoms of burnout, depression, and anxiety among athletes may involve shared risk pathways (Dalgleish et al., 2020).

Worry—particularly concerns regarding athletic performance, upcoming competitions, injury recovery, and selection outcomes—constitutes one of the primary psychological stressors faced by athletes (Ford et al., 2017). In prior research, GAD-2 (uncontrollable worry) has also been identified as a central symptom in the anxiety-depression network within the general population (Bai et al., 2021; Liu et al., 2025). For athletes, the majority of their pursuits involve striving for control—over their bodies, skills, performance, and environment. When worry becomes uncontrollable, it directly undermines athletes’ sense of mastery over competitive situations, thereby intensifying their psychological distress (Yang et al., 2024). Several sport-specific mechanisms may help explain why uncontrollable worry occupies such a central position among athletes. First, perfectionism is among the most robust personality-level predictors of psychological distress in athletes (Gonzalez-Hernandez et al., 2025). A meta-analysis by Hill and Curran (2016) showed that perfectionistic concerns (i.e., an excessive concern over mistakes, fear of negative evaluation, and a perceived discrepancy between personal standards and performance) are consistently associated with higher levels of burnout, and longitudinal evidence further indicates that such concerns prospectively predict increases in athlete burnout over time (Madigan et al., 2015). Athletes high in perfectionistic concerns impose on themselves standards that are often unattainable and continually monitor the gap between actual and expected performance (Stoeber and Otto, 2006; Stoeber, 2011); when this gap is perceived repeatedly, the self-evaluative process can readily escalate into chronic, uncontrollable worry (Hill et al., 2018; Xie et al., 2019). Second, fear of failure is also closely associated with worry in athletes (Taylor et al., 2023). Athletes tend to appraise failure as a threat that brings shame and a loss of social standing, so that worry about failure is frequent and difficult to dispel (Conroy et al., 2002). Unlike most achievement domains, failure for athletes is simultaneously tied to multiple high-stakes consequences, including athletic identity (Liu et al., 2025), evaluation by coaches and peers (Lervold et al., 2025), and, for collegiate athletes, the security of their athletic scholarships (Van Rheenen, 2013). When the consequences of failure are this multidimensional and difficult to reverse, worry becomes hard to switch off, turning what would otherwise be transient pre-competition worry into persistent, uncontrollable rumination (Nogueira et al., 2025).

Acceptance and Commitment Therapy (ACT) extends this account at the process level, offering an explanation for why athletes’ worries, once triggered, prove so resistant to dissipation. Within the ACT framework, worry persists in sport settings not because of what athletes think, but because of how they relate to their thoughts, a relational stance captured by the construct of cognitive fusion (Hayes et al., 2006). When an athlete becomes fused with thoughts such as “I cannot lose this match” or “I must perform flawlessly,” these cognitions are experienced as objective facts about the self and the world rather than as fleeting mental events open to observation. In this state, the individual is drawn toward suppression, avoidance, or repeated mental rehearsal as means of escaping the discomfort, a pattern known as experiential avoidance (Hayes et al., 1996). Yet avoidance paradoxically amplifies the psychological force of these thoughts, allowing worry to repeatedly recapture attentional resources and, in doing so, erode performance in the present moment (Gardner and Moore, 2012; Birrer et al., 2012).

As a hallmark feature of major depressive disorder (Otte et al., 2016), PHQ-2 (depressed mood) is closely linked to hopelessness, which the hopelessness theory posits as both an immediate antecedent and a sufficient precondition for depressive onset: when individuals develop negative expectations about the future and perceive themselves as incapable of altering their current circumstances, they experience intense feelings of hopelessness, which in turn directly precipitate depressive symptoms (Zhao et al., 2022). Research has demonstrated that, compared to the general population, athletes endure higher levels of psychological stress and are more susceptible to experiencing hopelessness (Beable et al., 2017). Several sport-specific pathways can amplify depressed mood in athletes. One of the most distinctively athletic sources of vulnerability is athletic identity foreclosure (Brewer and Petitpas, 2017), whereby an individual commits strongly to the single identity of “athlete” before having adequately explored academic, occupational, and other social roles. DeFreese and Shannon (2021) demonstrated that once this singular identity is highly internalized, any event that threatens the athletic identity, such as a serious injury or a slump in form, can directly shake an individual’s core sense of self-worth. Green and Weinberg (2001) likewise found that the stronger the athletic identity, the greater the psychological impact of injury. Under these conditions, a defeat or a spell of injury-related absence is no longer a localized setback but is experienced by the athlete as a global negation of “who I am,” which in turn develops into persistent and pervasive depressed mood (Brewer, 1993). In addition, the loss of motivation that follows injury or competitive defeat is likewise closely associated with depressed mood in athletes. Self-determination theory holds that when athletes’ basic psychological needs for autonomy, competence, and relatedness are repeatedly thwarted, their motivation declines along the self-determination continuum, regressing from intrinsic motivation to external regulation and ultimately to amotivation (Deci and Ryan, 2000; Vallerand and Losier, 1999); individuals in a state of amotivation often experience a loss of interest, a diminished sense of worth, and low mood, which are high-risk markers of depressed mood (Li et al., 2013; Wu et al., 2021). Because athletes’ training and competition are inherently goal-directed, recurrent injuries and competitive failures continually deprive them of opportunities for need satisfaction and goal attainment, leaving them persistently frustrated at the motivational level and thereby linking these experiences to depressed mood (Li et al., 2017). Putukian (2016) further noted that the helplessness and hopelessness induced by injury are important triggers of depressive symptoms in collegiate athletes. During major competition cycles, the phenomenon of “post-competition blues” in athletes may also produce short-term elevations in PHQ-2 symptoms. Drawing on an interpretative phenomenological analysis of four British female athletes at the Rio Olympics, Howells and Lucassen (2018) found that the negative affect experienced during this period stemmed primarily from a failure to meet established performance expectations and was further amplified by a sharp decline in media attention and a fall from “celebrity” status. This suggests that an imbalance in athletes’ sense of identity may contribute to a downturn in mood. Such a transient yet pervasive downturn may be a prototypical situational manifestation of the central position that PHQ-2 occupies in the network.

ABQ-E8 describes a state of physical and emotional exhaustion resulting from prolonged high-intensity training and competitive pressure. This symptom constitutes a core component of athlete burnout, corresponding to emotional exhaustion within the three-dimensional model of burnout. In existing comorbidity network studies, nodes related to emotional exhaustion often exhibit dense connections with symptoms of depression and anxiety, and in some studies have emerged as key bridge symptoms (Ernst et al., 2021; Wang et al., 2025). Compared to the general population, competitive athletes more frequently encounter situations of overtraining and insufficient recovery; sustained excessive physiological and psychological demands render them more vulnerable to chronic fatigue and energy depletion. This sense of exhaustion is frequently accompanied by diminished interest and reduced sense of accomplishment. Athletes may feel that their motivation exceeds their capacity, finding themselves unable to achieve established goals, which consequently engenders frustration and low self-esteem—experiences that closely parallel those of depressed mood. Consequently, elevated levels of physical and emotional exhaustion significantly increase the risk of developing depressive symptoms (Vasiliauskas and Rausch, 2024). The state of exhaustion captured by ABQ-E8 can be understood through the concept of overtraining syndrome (OTS). OTS refers to a sustained decline in athletic performance caused by a prolonged imbalance between training stress and recovery (Meeusen et al., 2013), and its clinical presentation overlaps substantially with that of depression, encompassing persistent fatigue, sleep disturbance, loss of interest, and depressed mood (Armstrong and VanHeest, 2002). This overlap may stem from a shared neurobiological basis, such as dysregulation of the hypothalamic–pituitary–adrenal (HPA) axis and elevated pro-inflammatory signaling, both of which have been reported in OTS and in major depression (Miller et al., 2009; Cadegiani and Kater, 2017; Smith, 2000). This common basis of inadequate recovery offers one explanation for why physical exhaustion occupies a central position in the network and forms its strongest connection with fatigue (PHQ-4). The two may reflect the same state of chronic under-recovery, thereby linking burnout and depression.

Furthermore, bridge expected influence (1-step) quantifies the bridging role of specific symptoms across different symptom clusters (burnout, depression, and anxiety), thereby identifying bridge symptoms that play critical mediating roles in cross-cluster transmission. Our findings revealed that PHQ-3 (sleep disturbance) was the bridge symptom with the highest strength, a finding consistent with previous research (Dong et al., 2024; Emami et al., 2025; Wang et al., 2025). For athletes, sleep is not merely rest but represents the most essential mode of recovery, playing a crucial role in physical restoration, hormonal regulation, cognitive function, and immune system functioning. Athletes may even require longer sleep duration than the general population (approximately 9 to 10 h) to achieve complete recovery (Bonnar et al., 2018). A recent meta-analysis confirmed that sleep insufficiency produces medium to large effect sizes of impairment across multiple athletic performance domains, including explosive power, speed, and skill control (Kong et al., 2025), thereby exacerbating burnout risk among athletes. Moreover, from a neurobiological perspective, emotion regulation relies on the coordinated functioning of the prefrontal-limbic system, with the ventromedial prefrontal cortex and anterior cingulate cortex serving as critical hubs that downregulate negative affective states by maintaining functional connections with the amygdala (Motzkin et al., 2015; Quirk and Beer, 2006). Sleep insufficiency or sleep deprivation is associated with heightened amygdala reactivity to negative stimuli and weakened functional connectivity between the amygdala and prefrontal regulatory regions, potentially reducing the efficiency of negative emotion regulation and increasing emotional reactivity (Goldstein and Walker, 2014). In anxiety and depressive disorders, neuroimaging evidence related to negative emotional processing biases and regulatory network abnormalities is also frequently observed (Etkin and Wager, 2007; Price and Drevets, 2010). These findings further support the critical bridging role of sleep disturbance in the interconnections among burnout, anxiety, and depressive symptoms.

Following the determination of the network importance of symptom nodes, the analysis of edge weights between symptoms also holds significant theoretical and practical value. Strongly associated symptom pairs indicate high temporal synchrony and interdependence between these symptoms; such close covariation implies that effective intervention targeting any one of these symptoms may facilitate concurrent improvement in associated symptoms. Our findings revealed that ABQ-E8 (Physical exhaustion) and PHQ-4 (fatigue), as well as ABQ-E2 (training fatigue) and PHQ-1 (Anhedonia), were the two most strongly associated symptom pairs. This finding suggests that athlete burnout and depression exhibit overlap at the symptom level, particularly with respect to energy depletion and anhedonia. Indeed, burnout is often defined as a state of energy exhaustion manifested by individuals under prolonged unmanageable stress (Bianchi and Schonfeld, 2018), and the characteristic symptoms of depression similarly include persistent fatigue and loss of interest in daily activities (Nixdorf et al., 2020). Our results are consistent with this overlap, suggesting that burnout may largely reflect a stress-induced depressive response.

Beyond describing this overlap, the specific configuration of these two edges points to a possible mechanistic pathway. Both of the strongest edges originate in the exhaustion dimension of burnout (ABQ-E2, ABQ-E8) and terminate in the somatic-motivational symptoms of depression (PHQ-4, PHQ-1) rather than in its cognitive-affective symptoms (e.g., worthlessness or low mood). This asymmetry suggests that, among athletes, burnout may be transmitted to depression primarily through a somatic-exhaustion pathway: prolonged training load combined with inadequate recovery keeps individuals in a sustained state of physical and energetic exhaustion, which manifests directly as low energy and fatigue (PHQ-4); and, as athletes’ capacity to invest effort in their sport and to derive pleasure from it is eroded, this exhaustion further manifests as diminished interest and pleasure (PHQ-1). This interpretation is reinforced by the central position of physical exhaustion (ABQ-E8) in the present network (EI = 1.11): its centrality makes physical exhaustion an energetic hub from which depressive symptoms may radiate outward. The pathway is also biologically plausible: overtraining without adequate recovery has been found to be associated with dysregulation of central serotonergic and dopaminergic signaling and can induce fatigue, reduced motivation, and anhedonia that closely resemble depressive symptoms (Meeusen et al., 2007, 2013). Building on this, stress-related elevations in pro-inflammatory signaling and HPA-axis dysregulation may further downregulate the mesolimbic dopamine reward circuit, thereby exacerbating anhedonia (Felger and Treadway, 2017; Miller et al., 2009). On this understanding, burnout and depression in athletes constitute an exhaustion-driven cascade, in which physical exhaustion serves as the proximal entry point to depressive fatigue and anhedonia.

The findings of the present study hold important practical implications for the assessment, prevention, and intervention of athlete mental health. First, the identified central symptoms exhibited the strongest associations with other symptoms within the symptom network and may play critical roles in the onset and maintenance of psychological problems. Therefore, designing targeted intervention programs centered on these central symptoms may trigger broader cascading effects, thereby enhancing overall intervention efficacy. Second, bridge symptoms, as important nodes connecting different symptom clusters, reflect potential risk pathways through which burnout, depression, and anxiety mutually transmit and influence one another. Prioritizing the monitoring and regulation of these bridge symptoms may help interrupt vicious cycles between different psychological problems, thereby preventing the onset and exacerbation of comorbidity. This provides coaches, sport psychologists, and sports medicine personnel with clear and specific intervention targets. Third, the results of the present study contribute to the construction of a more refined athlete mental health monitoring system. Compared to traditional total score-based screening approaches, attending to dynamic changes in specific symptom nodes enables earlier and more sensitive risk identification, thereby facilitating the timely provision of necessary psychological support for athletes on the verge of burnout or emotional distress. Finally, the findings of this study provide a theoretical foundation for developing more concise and efficient early screening instruments, facilitating the early identification and prevention of at-risk athletes.

## 5 Limitations

The present study also has several limitations, which at the same time point to fruitful directions for future research. First, we employed a cross-sectional design, and the resulting network was a regularized partial correlation network. Partial correlations reflect the conditional dependence between two symptoms after controlling for all other nodes, and as such they cannot in themselves establish the direction or causality of an association. Future research could use longitudinal panel networks and cross-lagged designs to examine the temporal ordering among symptoms, or employ ecological momentary assessment to characterize the dynamic covariation and time-lagged transmission of symptoms within everyday training and competition contexts. Such approaches would help convert the directional hypotheses suggested by cross-sectional edge weights into testable dynamic pathways and identify upstream symptoms that could serve as targets for intervention. Although the present study reported the data collection period, it did not systematically record the specific competitive phase that participants were in. Because the sample spanned multiple sports, participants were likely at different stages of their respective competitive seasons. Given that burnout, depression, and anxiety are sensitive to fluctuations in training load and competitive stress, this unrecorded heterogeneity may have acted as a confounder, influencing baseline symptom levels and the estimated network structure. Future studies should record competitive phase at the time of assessment and include it as a covariate or grouping variable, thereby distinguishing symptom-level interactions from season-related variation. Third, all three constructs were assessed solely through self-report questionnaires administered at a single time point and in a single format, which may introduce common method bias and artificially inflate the correlations among items. Future research could mitigate this by incorporating multi-method, multi-source data, such as clinician-administered interview ratings, informant reports from coaches or teammates, and objective measures of sleep and recovery based on physiological indices and actigraphy. Cross-validating subjective self-reports against objective indicators would both test the robustness of the network structure and help distinguish substantive symptom associations from spurious associations arising from method effects. Fourth, depressive and anxiety symptoms were measured using the PHQ-9 and GAD-7, respectively, both of which were developed for the general population. This may overestimate the connection between burnout and depression, as well as the prominence of somatic bridge symptoms such as sleep disturbance (PHQ-3). Future research could introduce sport-specific instruments (e.g., a sport-competition anxiety scale and sport-specific measures of depression or mood) and compare the resulting network structure with that obtained using generic instruments, in order to determine the extent to which symptom associations reflect genuine psychopathological comorbidity. Finally, the study sample consisted entirely of Chinese collegiate athletes, which limits the cultural and contextual generalizability of the findings. Future research should undertake cross-cultural and multi-sport replication studies, comparing differences in network structure across cultures, competitive levels (e.g., professional and elite), and types of sport.

## 6 Conclusion

Through network analysis, this study examined the interrelationships among athlete burnout, depression, and anxiety, identifying GAD-2 (uncontrollable worry), PHQ-2 (depressed mood), and ABQ-E8 (physical exhaustion) as the most central nodes with the highest centrality values. Concurrently, PHQ-3 (sleep disturbance) served a bridging function, effectively connecting different symptom clusters. Furthermore, network analysis identified two critical strongly connected pathways: the direct association between physical exhaustion and fatigue (ABQ-E8—PHQ-4), and the close connection between training fatigue and anhedonia (ABQ-E2—PHQ-1). These findings provide novel insights into the internal relationships among the three symptom domains, while also facilitating the development of more concise early screening instruments for the timely identification and prevention of at-risk athletes. Additionally, these results lay the foundation for developing individualized intervention programs tailored to athlete populations with different characteristics (e.g., sport type, sex) in future research.

## Data Availability

The original contributions presented in the study are included in the article/Supplementary material, further inquiries can be directed to the corresponding authors.

## Footnotes

1
https://www.wjx.cn/
