# Supplementary material for: Exploring the interrelationships between athlete burnout, depression, and anxiety: a network analysis approach
Source: Front Psychol. 2026 Jul 7;17:1845637. doi: 10.3389/fpsyg.2026.1845637 (PMC13384827; doi:10.3389/fpsyg.2026.1845637)
Supplement: Supplementary file 1 [file Supplementary_file_1.docx]

**Exploring the Interrelationships Between Athlete Burnout, Depression, and Anxiety: A Network Analysis Approach**

***Appendix A. Network Stability and Accuracy***


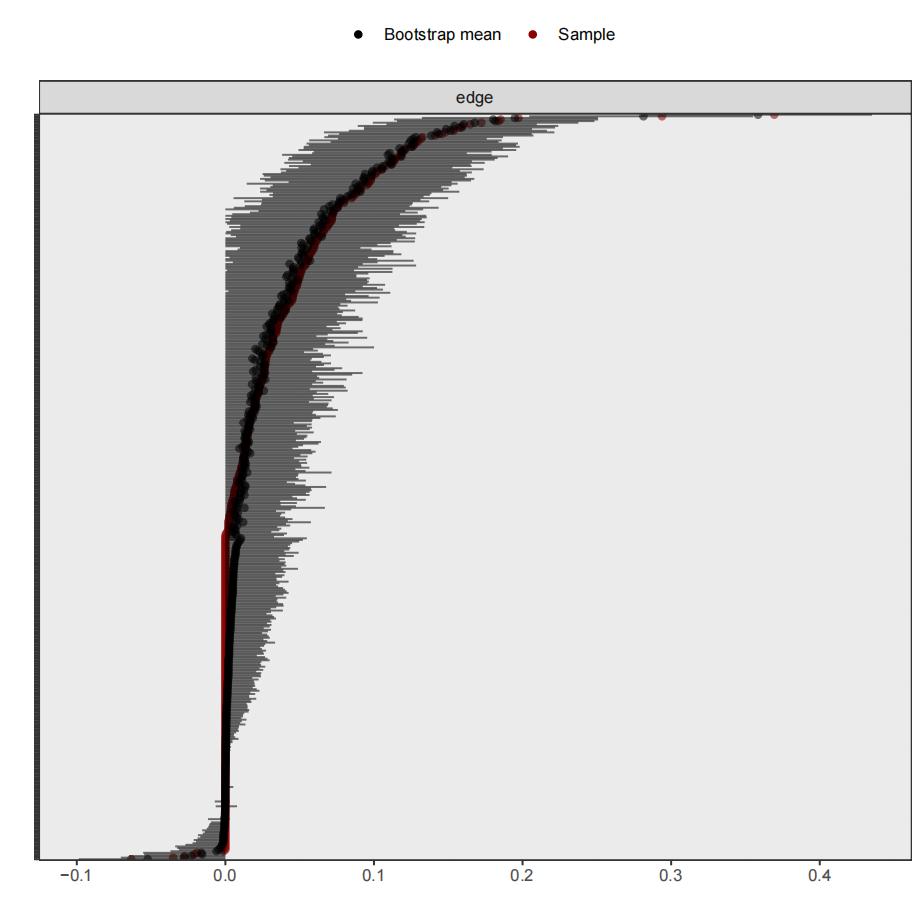


**Fig. S1.** Nonparametric bootstrapped confidence intervals of estimated edges. The red line represents the estimated edge, while the shaded area indicates the 95% bootstrap confidence interval.


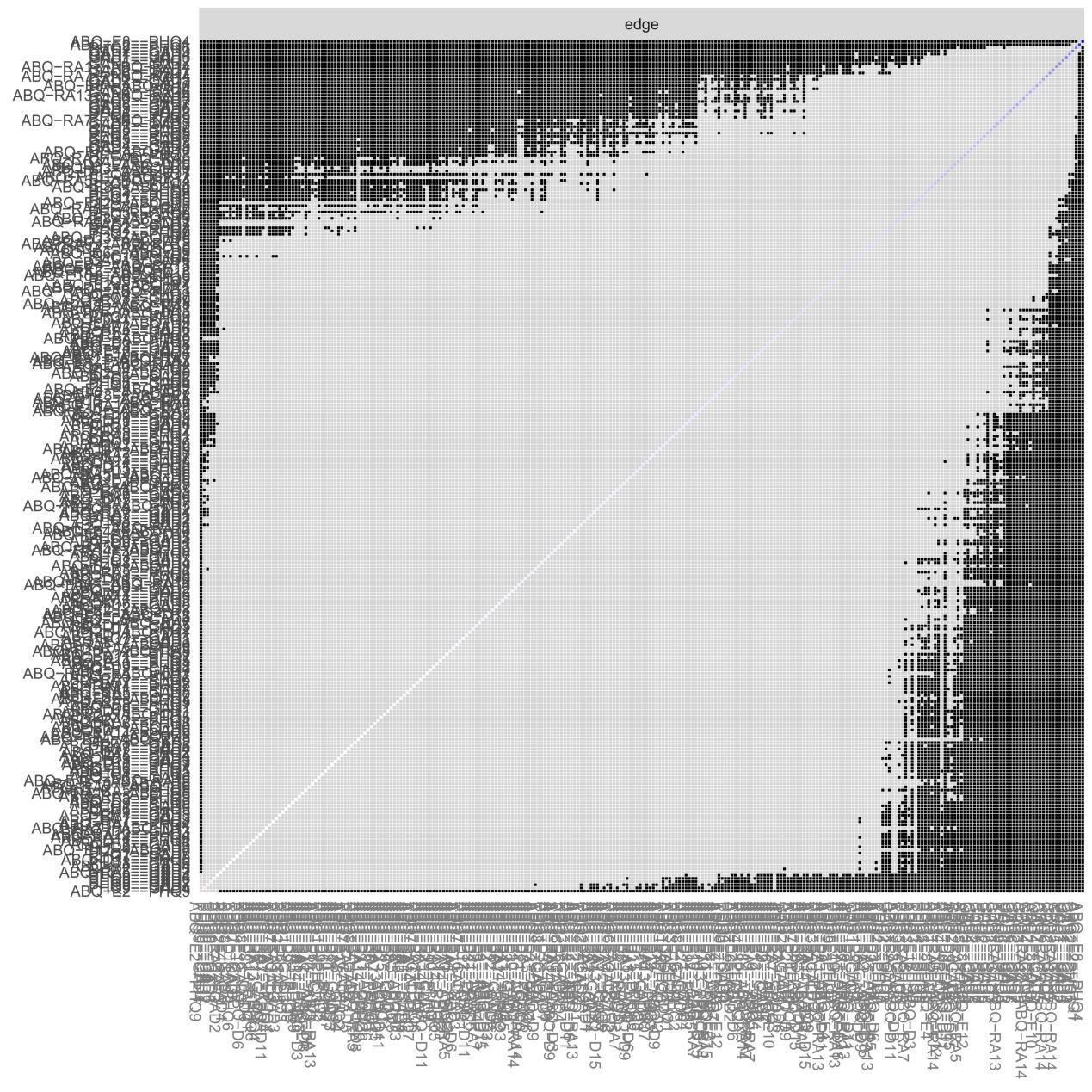


**Fig. S2.** Nonparametric bootstrapped difference test for edges. Grey boxes indicate no significant difference, whereas black boxes indicate a statistically significant difference (*p* < 0.05)

***Appendix B. Comparisons Based on Sex***

To formally test sex differences in network characteristics, we conducted the Network Comparison Test (NCT) using the NetworkComparisonTest package (version 2.2.1) in R (Van Borkulo et al., 2023). The NCT is a permutation-based procedure designed to evaluate differences between two independently estimated networks. All permutation tests were based on 1,000 permutations, and multiple comparison corrections were applied to the edge-specific tests.

we compared global strength, network structure, and individual edge weights between male and female groups. The network topologies of both groups exhibited highly similar structural characteristics. Comparison of global strength revealed a minimal and non-significant difference between males (12.57) and females (12.72), with S = 0.15 and p = 0.75. Likewise, the invariance test for edge weight distribution showed no significant gender difference (M = 0.22, p = 0.70) (see Figure s4)**.**


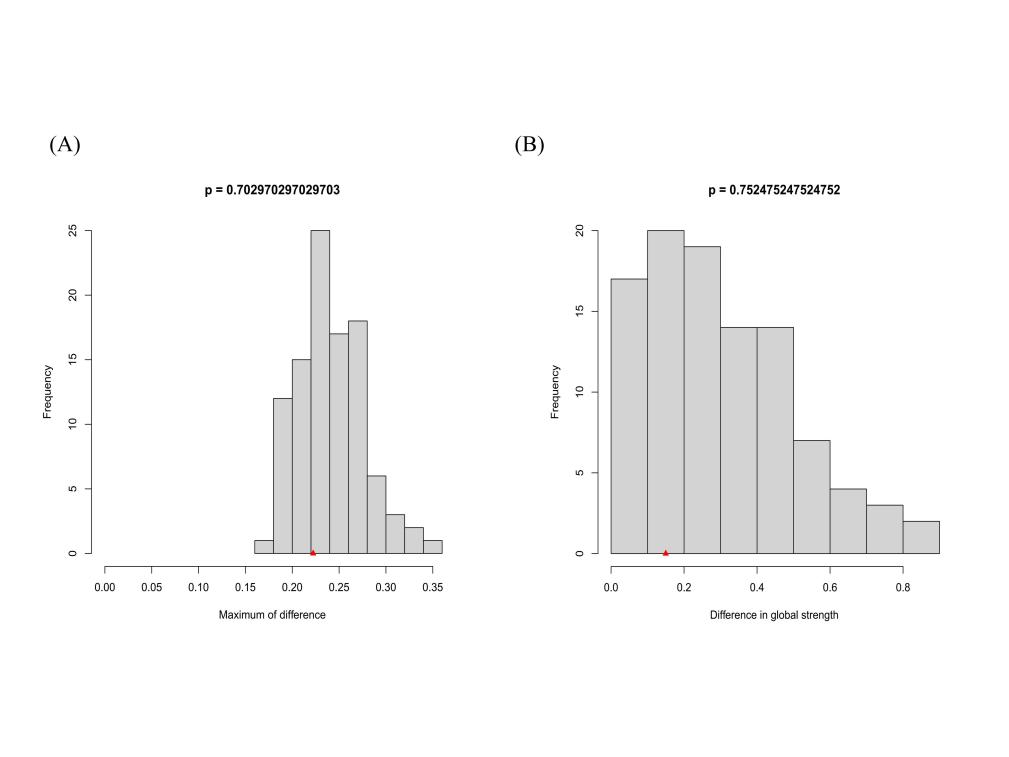


**Figure S4.** (A), the difference in global strength. (B), the maximum difference in edge strength.

***Appendix C. Expected influence (EI) and bridge expected influence (bEI) of all symptom nodes.***

**Table S1. Expected influence (EI) and bridge expected influence (bEI) of all symptom nodes.**

| **Node** | **Community** | **EI** | **bEI** |
| --- | --- | --- | --- |
| ABQ-E8 | Burnout | 1.109 | 0.414 |
| ABQ-E2 | Burnout | 0.692 | 0.334 |
| ABQ-E4 | Burnout | 0.696 | 0.306 |
| ABQ-E10 | Burnout | 0.652 | 0.124 |
| ABQ-E12 | Burnout | 0.757 | 0.175 |
| ABQ-RA1 | Burnout | 0.686 | 0.113 |
| ABQ-RA5 | Burnout | 0.822 | 0.250 |
| ABQ-RA7 | Burnout | 0.857 | 0.306 |
| ABQ-RA13 | Burnout | 0.704 | 0.095 |
| ABQ-RA14 | Burnout | 0.811 | 0.080 |
| ABQ-D3 | Burnout | 0.753 | 0.284 |
| ABQ-D6 | Burnout | 0.682 | 0.234 |
| ABQ-D9 | Burnout | 0.710 | 0.060 |
| ABQ-D11 | Burnout | 0.691 | 0.250 |
| ABQ-D15 | Burnout | 0.682 | 0.117 |
| PHQ-1 | Depression | 0.972 | 0.442 |
| PHQ-2 | Depression | 1.224 | 0.239 |
| PHQ-3 | Depression | 0.985 | 0.474 |
| PHQ-4 | Depression | 0.958 | 0.436 |
| PHQ-5 | Depression | 0.920 | 0.268 |
| PHQ-6 | Depression | 0.964 | 0.191 |
| PHQ-7 | Depression | 0.975 | 0.182 |
| PHQ-8 | Depression | 0.901 | 0.119 |
| PHQ-9 | Depression | 0.320 | -0.039 |
| GAD-1 | Anxiety | 0.984 | 0.241 |
| GAD-2 | Anxiety | 1.070 | 0.305 |
| GAD-3 | Anxiety | 0.932 | 0.159 |
| GAD-4 | Anxiety | 0.940 | 0.225 |
| GAD-5 | Anxiety | 1.015 | 0.188 |
| GAD-6 | Anxiety | 0.955 | 0.288 |
| GAD-7 | Anxiety | 0.954 | 0.190 |

*Note.* N = 1,226. EI = expected influence; bEI = bridge expected influence; both are reported as raw (non-standardized) values. Nodes are grouped by community (Burnout, Depression, Anxiety) and listed in their original item order within each community. ABQ = Athlete Burnout Questionnaire-15, with subscales E (emotional/physical exhaustion), RA (reduced sense of accomplishment), and D (devaluation); PHQ = Patient Health Questionnaire-9 (depression); GAD = Generalized Anxiety Disorder Scale-7 (anxiety).

***Appendix D. Weighted adjacency matrix.***

**Table S2. Weighted adjacency matrix.**

|  | 1 | 2 | 3 | 4 | 5 | 6 | 7 | 8 | 9 | 10 | 11 | 12 | 13 | 14 | 16 | 17 | 18 | 19 | 20 | 21 | 22 | 23 | 24 | 25 | 26 | 27 | 28 | 29 | 30 | 31 |
| --- | --- | --- | --- | --- | --- | --- | --- | --- | --- | --- | --- | --- | --- | --- | --- | --- | --- | --- | --- | --- | --- | --- | --- | --- | --- | --- | --- | --- | --- | --- |
| ABQ-E8 | 0.00 | 0.05 | 0.09 | 0.13 | 0.10 | 0.06 | 0.00 | 0.00 | 0.07 | 0.05 | 0.01 | 0.01 | 0.08 | 0.03 | 0.02 | 0.01 | 0.00 | 0.00 | 0.37 | 0.00 | 0.00 | 0.00 | 0.00 | 0.00 | 0.00 | 0.03 | 0.00 | 0.00 | 0.00 | 0.00 |
| ABQ-E2 | 0.05 | 0.00 | 0.06 | 0.00 | 0.07 | 0.00 | 0.00 | 0.02 | 0.03 | 0.00 | 0.02 | 0.05 | 0.06 | 0.01 | 0.00 | 0.29 | 0.00 | 0.00 | 0.00 | 0.00 | 0.00 | 0.00 | 0.00 | -0.06 | 0.03 | 0.02 | 0.00 | 0.00 | 0.00 | 0.04 |
| ABQ-E4 | 0.09 | 0.06 | 0.00 | 0.05 | 0.05 | 0.00 | 0.01 | 0.03 | 0.00 | 0.02 | 0.00 | 0.03 | 0.02 | 0.02 | 0.00 | 0.00 | 0.00 | 0.06 | 0.00 | 0.03 | 0.02 | 0.00 | 0.00 | 0.00 | 0.05 | 0.05 | 0.00 | 0.00 | 0.00 | 0.03 |
| ABQ-E10 | 0.13 | 0.00 | 0.05 | 0.00 | 0.10 | 0.05 | 0.02 | 0.04 | 0.06 | 0.02 | 0.00 | 0.01 | 0.00 | 0.00 | 0.05 | 0.01 | 0.05 | 0.00 | 0.00 | 0.05 | 0.00 | 0.01 | 0.00 | 0.00 | 0.00 | 0.00 | 0.00 | 0.00 | 0.00 | 0.00 |
| ABQ-E12 | 0.10 | 0.07 | 0.05 | 0.10 | 0.00 | 0.00 | 0.00 | 0.05 | 0.01 | 0.00 | 0.08 | 0.00 | 0.04 | 0.02 | 0.07 | 0.00 | 0.01 | 0.04 | 0.00 | 0.00 | 0.00 | 0.00 | 0.00 | 0.00 | 0.00 | 0.02 | 0.02 | 0.05 | 0.04 | 0.00 |
| ABQ-RA1 | 0.06 | 0.00 | 0.00 | 0.05 | 0.00 | 0.00 | 0.10 | 0.05 | 0.06 | 0.16 | 0.01 | 0.02 | 0.07 | 0.00 | 0.00 | 0.00 | 0.01 | 0.00 | 0.00 | 0.03 | 0.00 | 0.00 | 0.00 | 0.06 | 0.00 | 0.00 | 0.00 | 0.00 | 0.00 | 0.00 |
| ABQ-RA5 | 0.00 | 0.00 | 0.01 | 0.02 | 0.00 | 0.10 | 0.00 | 0.08 | 0.07 | 0.09 | 0.03 | 0.03 | 0.00 | 0.07 | 0.07 | 0.04 | 0.00 | 0.02 | 0.00 | 0.01 | 0.02 | 0.01 | 0.01 | 0.01 | 0.00 | 0.00 | 0.06 | 0.01 | 0.01 | 0.05 |
| ABQ-RA7 | 0.00 | 0.02 | 0.03 | 0.04 | 0.05 | 0.05 | 0.08 | 0.00 | 0.12 | 0.15 | 0.00 | 0.01 | 0.00 | 0.00 | 0.00 | 0.01 | 0.02 | 0.05 | 0.00 | 0.00 | 0.01 | 0.04 | 0.00 | 0.00 | 0.03 | 0.04 | 0.00 | 0.03 | 0.01 | 0.03 |
| ABQ-RA13 | 0.07 | 0.03 | 0.00 | 0.06 | 0.01 | 0.06 | 0.07 | 0.12 | 0.00 | 0.13 | 0.00 | 0.00 | 0.03 | 0.00 | 0.03 | 0.00 | 0.00 | 0.00 | 0.00 | 0.01 | 0.00 | 0.03 | 0.00 | 0.05 | 0.00 | 0.00 | 0.00 | 0.00 | 0.00 | 0.00 |
| ABQ-RA14 | 0.05 | 0.00 | 0.02 | 0.02 | 0.00 | 0.16 | 0.09 | 0.15 | 0.13 | 0.00 | 0.01 | 0.00 | 0.03 | 0.01 | 0.06 | 0.02 | 0.00 | 0.02 | 0.00 | 0.02 | 0.00 | 0.01 | 0.00 | 0.01 | 0.00 | 0.00 | 0.00 | 0.00 | 0.00 | 0.00 |
| ABQ-D3 | 0.01 | 0.02 | 0.00 | 0.00 | 0.08 | 0.01 | 0.03 | 0.00 | 0.00 | 0.01 | 0.00 | 0.07 | 0.10 | 0.07 | 0.07 | 0.00 | 0.02 | 0.01 | 0.00 | 0.01 | 0.03 | 0.01 | 0.02 | 0.00 | 0.02 | 0.02 | 0.01 | 0.02 | 0.05 | 0.03 |
| ABQ-D6 | 0.01 | 0.05 | 0.03 | 0.01 | 0.00 | 0.02 | 0.03 | 0.01 | 0.00 | 0.00 | 0.07 | 0.00 | 0.07 | 0.10 | 0.06 | 0.01 | 0.05 | 0.00 | 0.00 | 0.01 | 0.00 | 0.00 | 0.00 | 0.00 | 0.01 | 0.05 | 0.00 | 0.03 | 0.02 | 0.01 |
| ABQ-D9 | 0.08 | 0.06 | 0.02 | 0.00 | 0.04 | 0.07 | 0.00 | 0.00 | 0.03 | 0.03 | 0.10 | 0.07 | 0.00 | 0.06 | 0.10 | 0.00 | 0.00 | 0.00 | 0.00 | 0.04 | 0.00 | 0.00 | 0.01 | 0.00 | 0.00 | 0.00 | 0.00 | 0.00 | 0.00 | 0.00 |
| ABQ-D11 | 0.03 | 0.01 | 0.02 | 0.00 | 0.02 | 0.00 | 0.07 | 0.00 | 0.00 | 0.01 | 0.07 | 0.10 | 0.06 | 0.00 | 0.05 | 0.00 | 0.00 | 0.03 | 0.06 | 0.02 | 0.00 | 0.02 | 0.00 | 0.00 | 0.02 | 0.01 | 0.01 | 0.04 | 0.02 | 0.02 |
| ABQ-D15 | 0.02 | 0.00 | 0.00 | 0.05 | 0.07 | 0.00 | 0.07 | 0.00 | 0.03 | 0.06 | 0.07 | 0.06 | 0.10 | 0.05 | 0.00 | 0.01 | 0.04 | 0.00 | 0.00 | 0.03 | 0.03 | 0.00 | 0.00 | 0.00 | 0.00 | 0.00 | 0.00 | 0.00 | 0.00 | 0.00 |
| PHQ1 | 0.01 | 0.29 | 0.00 | 0.01 | 0.00 | 0.00 | 0.04 | 0.01 | 0.00 | 0.02 | 0.00 | 0.01 | 0.00 | 0.00 | 0.01 | 0.00 | 0.16 | 0.01 | 0.07 | 0.09 | 0.04 | 0.07 | 0.09 | 0.00 | 0.00 | 0.00 | 0.02 | 0.00 | 0.00 | 0.00 |
| PHQ2 | 0.00 | 0.00 | 0.00 | 0.05 | 0.01 | 0.01 | 0.00 | 0.02 | 0.00 | 0.00 | 0.02 | 0.05 | 0.00 | 0.00 | 0.04 | 0.16 | 0.00 | 0.11 | 0.13 | 0.11 | 0.20 | 0.13 | 0.07 | 0.07 | 0.00 | 0.03 | 0.00 | 0.00 | 0.00 | 0.00 |
| PHQ3 | 0.00 | 0.00 | 0.06 | 0.00 | 0.04 | 0.00 | 0.02 | 0.05 | 0.00 | 0.02 | 0.01 | 0.00 | 0.00 | 0.03 | 0.00 | 0.01 | 0.11 | 0.00 | 0.04 | 0.09 | 0.08 | 0.11 | 0.08 | 0.00 | 0.00 | 0.03 | 0.00 | 0.05 | 0.03 | 0.08 |
| PHQ4 | 0.37 | 0.00 | 0.00 | 0.00 | 0.00 | 0.00 | 0.00 | 0.00 | 0.00 | 0.00 | 0.00 | 0.00 | 0.00 | 0.06 | 0.00 | 0.07 | 0.13 | 0.04 | 0.00 | 0.05 | 0.05 | 0.10 | 0.09 | 0.00 | 0.00 | 0.00 | 0.00 | 0.00 | 0.00 | 0.00 |
| PHQ5 | 0.00 | 0.00 | 0.03 | 0.05 | 0.00 | 0.03 | 0.01 | 0.00 | 0.01 | 0.02 | 0.01 | 0.01 | 0.04 | 0.02 | 0.03 | 0.09 | 0.11 | 0.09 | 0.05 | 0.00 | 0.09 | 0.06 | 0.10 | 0.06 | 0.00 | 0.00 | 0.00 | 0.00 | 0.00 | 0.00 |
| PHQ6 | 0.00 | 0.00 | 0.02 | 0.00 | 0.00 | 0.00 | 0.02 | 0.01 | 0.00 | 0.00 | 0.03 | 0.00 | 0.00 | 0.00 | 0.03 | 0.04 | 0.20 | 0.08 | 0.05 | 0.09 | 0.00 | 0.15 | 0.12 | 0.05 | 0.03 | 0.00 | 0.03 | 0.00 | 0.00 | 0.00 |
| PHQ7 | 0.00 | 0.00 | 0.00 | 0.01 | 0.00 | 0.00 | 0.01 | 0.04 | 0.03 | 0.01 | 0.01 | 0.00 | 0.00 | 0.02 | 0.00 | 0.07 | 0.13 | 0.11 | 0.10 | 0.06 | 0.15 | 0.00 | 0.11 | 0.06 | 0.02 | 0.01 | 0.00 | 0.01 | 0.00 | 0.00 |
| PHQ8 | 0.00 | 0.00 | 0.00 | 0.00 | 0.00 | 0.00 | 0.01 | 0.00 | 0.00 | 0.00 | 0.02 | 0.00 | 0.01 | 0.00 | 0.00 | 0.09 | 0.07 | 0.08 | 0.09 | 0.10 | 0.12 | 0.11 | 0.00 | 0.12 | 0.04 | 0.01 | 0.00 | 0.02 | 0.00 | 0.00 |
| PHQ9 | 0.00 | -0.06 | 0.00 | 0.00 | 0.00 | 0.06 | 0.01 | 0.00 | 0.05 | 0.01 | 0.00 | 0.00 | 0.00 | 0.00 | 0.00 | 0.00 | 0.07 | 0.00 | 0.00 | 0.06 | 0.05 | 0.06 | 0.12 | 0.00 | 0.00 | -0.02 | 0.00 | -0.03 | 0.00 | -0.02 |
| GAD1 | 0.00 | 0.03 | 0.05 | 0.00 | 0.00 | 0.00 | 0.00 | 0.03 | 0.00 | 0.00 | 0.02 | 0.01 | 0.00 | 0.02 | 0.00 | 0.00 | 0.00 | 0.00 | 0.00 | 0.00 | 0.03 | 0.02 | 0.04 | 0.00 | 0.00 | 0.13 | 0.12 | 0.18 | 0.18 | 0.07 |
| GAD2 | 0.03 | 0.02 | 0.05 | 0.00 | 0.02 | 0.00 | 0.00 | 0.04 | 0.00 | 0.00 | 0.02 | 0.05 | 0.00 | 0.01 | 0.00 | 0.00 | 0.03 | 0.03 | 0.00 | 0.00 | 0.00 | 0.01 | 0.01 | -0.02 | 0.13 | 0.00 | 0.19 | 0.06 | 0.14 | 0.10 |
| GAD3 | 0.00 | 0.00 | 0.00 | 0.00 | 0.02 | 0.00 | 0.06 | 0.00 | 0.00 | 0.00 | 0.01 | 0.00 | 0.00 | 0.01 | 0.00 | 0.02 | 0.00 | 0.00 | 0.00 | 0.00 | 0.03 | 0.00 | 0.00 | 0.00 | 0.12 | 0.19 | 0.00 | 0.10 | 0.13 | 0.13 |
| GAD4 | 0.00 | 0.00 | 0.00 | 0.00 | 0.05 | 0.00 | 0.01 | 0.03 | 0.00 | 0.00 | 0.02 | 0.03 | 0.00 | 0.04 | 0.00 | 0.00 | 0.00 | 0.05 | 0.00 | 0.00 | 0.00 | 0.01 | 0.02 | -0.03 | 0.18 | 0.06 | 0.10 | 0.00 | 0.10 | 0.13 |
| GAD5 | 0.00 | 0.00 | 0.00 | 0.00 | 0.04 | 0.00 | 0.01 | 0.01 | 0.00 | 0.00 | 0.05 | 0.02 | 0.00 | 0.02 | 0.00 | 0.00 | 0.00 | 0.03 | 0.00 | 0.00 | 0.00 | 0.00 | 0.00 | 0.00 | 0.18 | 0.14 | 0.13 | 0.10 | 0.00 | 0.11 |
| GAD6 | 0.00 | 0.04 | 0.03 | 0.00 | 0.00 | 0.00 | 0.05 | 0.03 | 0.00 | 0.00 | 0.03 | 0.01 | 0.00 | 0.02 | 0.00 | 0.00 | 0.00 | 0.08 | 0.00 | 0.00 | 0.00 | 0.00 | 0.00 | -0.02 | 0.07 | 0.10 | 0.13 | 0.13 | 0.11 | 0.00 |
| GAD7 | 0.00 | 0.01 | 0.05 | 0.00 | 0.00 | 0.00 | 0.00 | 0.03 | 0.00 | 0.00 | 0.02 | 0.04 | 0.00 | 0.00 | 0.00 | 0.00 | 0.00 | 0.06 | 0.00 | 0.00 | 0.00 | 0.00 | 0.00 | -0.03 | 0.07 | 0.16 | 0.11 | 0.14 | 0.16 | 0.13 |

# **References**

Van Borkulo CD, Van Bork R, Boschloo L, et al. (2023) Comparing network structures on three aspects: A permutation test. *Psychological Methods* 28(6): 1273–1285.
